# Multiscale Supervised Classification of Point Clouds with Urban and Forest Applications

**DOI:** 10.3390/s19204523

**Published:** 2019-10-17

**Authors:** Carlos Cabo, Celestino Ordóñez, Fernando Sáchez-Lasheras, Javier Roca-Pardiñas, Javier de Cos-Juez

**Affiliations:** 1 Department of Mining Exploitation and Prospecting, University of Oviedo, 33003 Oviedo, Spain; carloscabo.uniovi@gmail.com (C.C.);; 2 Department of Applied Mathematics, University of Oviedo, 33003 Oviedo Spain; sanchezfernando@uniovi.es; 3 Department of Statistics and Operations Research, University of Vigo, 36310 Vigo, Spain; roca@uvigo.es

**Keywords:** point cloud, multiscale analysis, supervised classification

## Abstract

We analyze the utility of multiscale supervised classification algorithms for object detection and extraction from laser scanning or photogrammetric point clouds. Only the geometric information (the point coordinates) was considered, thus making the method independent of the systems used to collect the data. A maximum of five features (input variables) was used, four of them related to the eigenvalues obtained from a principal component analysis (PCA). PCA was carried out at six scales, defined by the diameter of a sphere around each observation. Four multiclass supervised classification models were tested (linear discriminant analysis, logistic regression, support vector machines, and random forest) in two different scenarios, urban and forest, formed by artificial and natural objects, respectively. The results obtained were accurate (overall accuracy over 80% for the urban dataset, and over 93% for the forest dataset), in the range of the best results found in the literature, regardless of the classification method. For both datasets, the random forest algorithm provided the best solution/results when discrimination capacity, computing time, and the ability to estimate the relative importance of each variable are considered together.

## 1. Introduction

Over the last three decades, there has been a proliferation of techniques for data collection, including geospatial data. At the same time, new mathematical methods to process those data have been developed, such as data mining, machine learning, big data, and, lately, deep learning. 3D point clouds, a type of geospatial data very widespread nowadays, are essentially massive files of point coordinates (X,Y,Z) that provide a discrete representation of the measured object in a Cartesian coordinate system. A significant problem with 3D point clouds is that they are unstructured collections of data, and this makes it necessary to process them in order to extract the information of interest. There are different methods for feature detection and extraction from 3D point clouds, although they can be divided into two main groups: direct (also called rules-driven) and indirect methods (data-driven methods). Direct methods are explicitly developed to solve a particular problem; for instance, in an urban environment, to extract roads [1,2,3,4], buildings [5,6,7], pole-like objects such as lampposts or trees [8,9], or traffic signs [10,11], but normally only one type of object per algorithm. They consist of a set of rules that take into account the geometric characteristics of the objects to be extracted that distinguish them from other objects in the scene. One of the advantages of these methods is that they require very little intervention by the users, beyond having to tune some parameters that affect the solution (e.g., voxel size, threshold distance between points for object segmentation, etc.). However, they may be difficult to implement in an algorithm and, as mentioned, they are normally devoted to extract a particular category of objects from the point cloud, not a group of them.

Indirect methods are those based on the application of machine learning techniques (specifically classification methods) to a set of features or explanatory variables that have been constructed from the point coordinates. They are usually easier to implement than direct methods since the effort is not put into devising the algorithm but into defining the features (explanatory variables) and selecting an adequate model among the different possibilities. They can be used to extract one or more types of objects at once, but a disadvantage is that they require a training procedure. In addition, they can be less accurate than direct methods in some cases.

Among indirect classification methods, there is also a subdivision into two categories: supervised and not supervised methods [12]. Supervised classification methods require user intervention to train the model, but this is not normally very time consuming for point cloud with the help of suitable software and, in return, they usually provide better solutions than unsupervised classification methods (see [13,14,15,16] as examples of the application of supervised classifications methods to 3D point clouds). Overfitting is a drawback associated to supervised classification, as it provides an accuracy in the training sample that is far above the accuracy that can be obtained in independent test samples. Deep learning for 3D point cloud classification and segmentation is an active research area at this moment that can displace conventional methods in a short time. With few exceptions [17], most of the deep learning approaches require transforming the point cloud into images or voxel meshes before feature learning using 2D (3D) convolutional neural networks [18]. However, there is still few literatures concerning deep learning for point segmentation and classification using aerial or terrestrial (static or mobile) laser scanning [19,20], and the results reported for now do not improve the best results obtained using rule-based methods or feature extraction combined with machine learning algorithms [21]. As shown in [22], a combination of convolutional neural networks with 3D point cloud features can improve the results obtained independently.

Unsupervised do not provide the solution in a training set to estimate the parameters of the mathematical model, so they need to look for similarities between the values of the different features in order to construct the clusters. Some algorithms require the user to preset the number of clusters in the data in advance, while others can make an estimation of the optimal number of clusters.

In this work, we are concerned with supervised classification methods, as we want to accurately extract some specific categories of objects from point clouds of different scenarios using the same algorithm, even at the expense of having to create training sets from the point clouds. In particular, we deal with a multiscale classification algorithm that assumes that the features defined for any observation (point) provide different information depending on the size of the neighborhood (the scale) around that observation. A reference work of multiscale supervised classification in geosciences is [23], whose algorithm is implemented in Cloud Compare, a free license software for point cloud processing and visualization that is widespread among users. The algorithm works directly with the point coordinates (neither the intensity nor the color) and allows some degree of variability in the class characteristics. The idea is that, depending on the scale, the cloud around a point can look like a line (1D object), a plane surface (2D object), or a volume (3D object). While this algorithm was initially developed to discriminate between two classes, in this work, we extend the algorithm to the multiclass problem. In addition, different features are used to train the models.

In summary, the most relevant aspects of this work are: the comparison of several multiclass machine learning algorithms for point cloud segmentation, the analysis of their performance for two kind of problems (urban and forest inventory), and the analysis of the influence of the scales and the extracted features in the results.

The paper is structured as follows: Section 2.1 focused on the definition of the features at different scales to be introduced in the models. Section 2.2 makes a brief explanation of the four classification techniques used in the case studies. In Section 3, we apply those classification methods to extract five classes of objects from a point cloud in an urban environment and three classes in a forestry area. The discussion of the results is given in Section 4. Finally, our conclusions are summarized in Section 5.

## 2. Methodology

### 2.1. Feature Extraction

A key step in the supervised classification analysis is to establish the features to be used in the model. In a multiscale method, those features are extracted at different scales, which tends to improve the results when only a single scale is used, as it has been stated in several works [24,25,26,27]. This is because a region of the cloud around an observation can look like a 1D, 2D, or 3D object depending on the size of that region [23]. Moreover, the multiscale strategy allows some degree of variability and heterogeneity in the characteristics of the different objects. For object detection and extraction from 3D point clouds, it is quite common to use the eigenvalues of a principal component analysis or some algebraic expressions relating them. These eigenvalue-based features have proven to be useful to describe the local geometry of the points. Examples of the application of this technique can be found in [15,27]. An analysis of the accuracy and robustness of the geometric features extracted from 3D point clouds was performed in [28].

Given any point $p_{i}=\left(X_{i},Y_{i},Z_{i}\right)$ of the point cloud, eigenvalues and eigenvectors are obtained through an eigendecomposition of the covariance matrix Σ:

(1) $$\Sigma =\frac{1}{N}\sum_{i=1}^{N}{\left(p_{i}-\bar{p}\right)}^{T}\left(p_{i}-\bar{p}\right)=V\Lambda V^{T},$$

where λ1>λ2>λ3 are the eigenvalues, and V a matrix which columns are the corresponding eigenvectors.

The relationship between the values of the eigenvalues λ1,λ2,λ3 at a point is related to the local geometry at that point [28]:

- A linear 1D structure when λ1≥λ2,λ3;
- A planar 2D structure when λ1,λ2≥λ3;
- A volumetric 3D structure when λ1≈λ2≈λ3.

In this study, we used five features at different scales as input variables (Table 1).

Z range for each point is calculated using the points in a vertical column of a specific section (scale) around that point. In order to avoid the negative effect of outliers, instead of using the Z coordinate ranges, we used the range between the 5th and 95th percentiles.

As will be seen below, these five variables allow to obtain an accurate classification of the point clouds in different categories, both for natural and artificial objects. Initially, artificial objects are expected to produce better results than natural objects in 3D point classification, as they are more homogeneous.

The scales are determined by the diameters of spheres around each point used to perform the PCA according to Equation (1) (so *N* in Equation (1) would be the number of points in that sphere). The five features calculated for the points in each sphere were assigned to the point in the center.

### 2.2. Classification Methods

Many classification methods have been described in the literature, but we choose to use four, according to their simplicity and proven discrimination capacity: linear discriminant analysis (LDA), multiclass logistic regression (LR), multiclass support vector machines (SVM), and random forest (RF). Although they are well-known methods that have been described and applied many times before, in the following sections we provide a brief summary of each of them, in order to highlight their fundamentals and assumptions.

#### 2.2.1. Linear Discriminant Analysis

LDA is, as its name indicates, a linear transformation that computes the directions of the axis that maximize the separation between multiple classes [29,30]. The data points are projected onto these directions. For a set of observations $X=(x_{1},x_{2},\ldots ,x_{n})$; $x_{i}\in R^{p}$ (in this study, p= number of scales and n= number of points), this can be accomplished by maximizing the ratio between the within-class $\left(S_{w}\right)$ and between-class $\left(S_{b}\right)$ scatter matrices, $R=\frac{S_{b}}{S_{w}}$, where

(2) $$S_{w}=\sum_{j=1}^{C}\sum_{i=1}^{n_{i}}\left(x_{ij}-\mu_{j}\right){\left(x_{ij}-\mu_{j}\right)}^{T};S_{b}=\frac{1}{C}\sum_{j=1}^{C}\left(\mu_{j}-\mu \right){\left(\mu_{j}-\mu \right)}^{T},$$

$\mu_{j}$ is the mean value for class *j*, $n_{i}$ is the number of elements in class *i*, and μ is the mean value for all the classes.

By maximizing *R*, the algorithm tries to assign elements to each class so that these are as homogeneous as possible while their means are as far apart as possible.

If $S_{w}$ is nonsingular, the solution is given by the eigenvectors of $S_{w}^{-1}S_{b}$ corresponding to the largest C−1 eigenvalues. These eigenvectors represent the directions of maximum separation between classes. LDA assumes that the features (input or explanatory variables) are continuous and normally distributed, while the dependent variable is categorical.

#### 2.2.2. Logistic Regression

Multinomial logistic regression [31] is in some way similar to LDA, since it also establishes a linear transformation between the output variable and input variables. However, the linear transformation is not between the output and the input variables, but between the input variables and the odds of the output categorical variable. In addition, input variables do not need to be continuous and normally distributed. They do not even have to be independent. The result is not the class for a category but the probability of an observation belonging to it.

The mathematical model for the multiclass logistic regression can be expressed as follows:

(3) $$ln\frac{P(Y=i)}{P(Y=C)}=\alpha_{i}+\sum_{j=1}^{p}\beta_{j}x_{j};i=1,\ldots ,C-1,$$

where *Y* represents the output variable (a categorical variable representing the class), $\alpha_{i}$ and $\beta {}_{j}$ are the coefficients of the model, and $x_{1},x_{2},\cdots ,x_{p}$ are the covariates. This means that a multiclass logistic regression model for C classes is equivalent to C-1 binary models considering one of the classes a reference (class C in Equation (3)). The rest of the classes are separately regressed against the reference. From (3) it follows that

(4) $$P\left(Y=i\right)=\frac{e^{\alpha_{i}+\sum_{j=1}^{j=p}\beta_{j}X_{j}}}{1+e^{\alpha_{i}+\sum_{j=1}^{j=p}\beta_{j}X_{j}}}.$$

Finally, each observation is assigned to the class of maximum probability.

#### 2.2.3. Support Vector Machines

Essentially, SVM algorithm [32] maps the original finite-dimensional space into a much higher-dimensional space where the boundaries between classes are hyperplanes of the form wX+b=0, where w represents the normal vector to the hyperplane and *b* represents the offset. SVM search for the maximum-margin hyperplane, that is, the hyperplane for which the distance to the closest observations of each class is maximized. In fact, the maximum-margin hyperplane lies halfway between two parallel hyperplanes (margin hyperplanes) that separate the two classes. The distance between these two planes, that must be maximum, is called margin. For a hypothetical perfectly separable case, no observation may lie between the margin hyperplanes. In a nonperfectly separable situation, the margin is "soft", which means that classification errors ξ are admitted and have to be minimized.

SVM is formulated as the following minimization problem with restrictions

$$\begin{array}{l} \min_{w,b}\frac{1}{2}{∥w∥}^{2}+C\sum_{i=1}^{n}\xi_{i}\\ y_{i}\left(wx_{i}+b\right)\geq 1-\xi_{i}\\ \xi_{i}\geq 0. \end{array}$$

The solution to this optimization problem is a linear combination of a subset of the original observations located near the margin hyperplanes, $w=\sum_{i=1}^{n}\alpha_{i}x_{i}$, named support vectors, that completely determine them (hence their name). *C* is a parameter that controls the generalization ability of an SVM.

Although they may seem very different, SVM resembles LR as both solve the same optimization problem, although with different loss functions. However, SVM is not a probabilistic method, so it directly assigns a class to an observation, not a probability.

Multiclass support vector machines is an extension of the original binary classifier to problems with more than two classes. Several strategies have been proposed [33], although a typical solution consists of solving several binary problems, such as with the “one-against-all” or “one-against-one” approaches. The first one constructs *C* binary classifiers, while the second constructs C(C−1)/2 classifiers, *C* being the number of classes.

#### 2.2.4. Random Forest

Random forests are assemblies of classification orregression trees (CART) [34,35] that use the bootstrap aggregated ensemble method to combine them and reduce the variance. This method consists of building multiple decision trees by resampling with replacement of the data and averaging the prediction. CART are built through a recursive binary partitioning, that involves iteratively splitting the data into subsets according to some rules. Then, the resulting subsets are split into two new datasets and so on until no more splits can be obtained according to some criterion. In each partition, the objective is to obtain a pair of homogeneous subsets. If this procedure is represented in a graphic from the top (initial node or dataset) to the bottom joining each subset (son node) by means lines (branches), then it resembles a tree. One positive characteristic of this machine learning method, compared with other methods such as LDA, LR, or SVM, is that it is easy to understand and to interpret, as it is close to human thinking.

There are different partition metrics or cost functions to evaluate splits. Specifically, CART use the Gini index of impurity, a measure of how mixed the classes are in the groups after a split. The minimum value is 0.0 (for a perfect separation) and the maximum value gets close to 1. For a set of items with *C* classes, the Gini index is

(5) $$G=\sum_{i=1}^{C}p_{i}\sum_{k\neq i}^{}p_{k}=1-\sum_{t=1}^{C}p_{i}^{2},$$

where $p_{i}$ is the fraction of items labeled with class *i* in the set, and $\sum_{k\neq i}p_{k}=1-p_{i}$ is the fraction of items labeled with a class different to *i*. From all the possible splits dictated by the data, the one with the smallest aggregated Gini index is selected.

One way to measure the relative importance of each input variable is to calculate the mean of the Gini index decrease throughout the tree. For each variable, the *G* is calculated and accumulated each time that variable is chosen to split a node. Finally, the sum is divided by the number of tress in the forest to calculate the average value.

### 2.3. Evaluation of the Results

To evaluate the performance of the different models, five metrics have been used: precision, recall, and F1 score for each class; and overall accuracy and Kappa coefficient for all classes. Precision measures the proportion of points classified as positives. Recall measures the proportion of positives that are real positive. F1 score is a metric that combines precision and recall. The strategy to calculate these metrics is one-versus-all, this means that any one of the metrics for each class is calculated against all the samples for the rest of the classes. Overall accuracy measures the proportion of points correctly classified, independently of the classes, and Kappa coefficient tells us how good the classification is compared to random assignment.

(6) $$\begin{array}{c} \mathrm{Precision}=\frac{TP}{TP+FP};\;\mathrm{Recall}=\frac{TP}{TP+FN};\;F1=2\frac{\mathrm{precision}\cdot \mathrm{recall}}{\mathrm{precision}+\mathrm{recall}}.\\ TP:\mathrm{true}\;\mathrm{positive};\;FP:\mathrm{false}\;\mathrm{positive};\;FN:\mathrm{false}\;\mathrm{negative}. \end{array}$$

The four supervised classification methods were trained with the same dataset and applied to the same test sample. The training dataset was balanced, so approximately the same number of points were stored for each class.

## 3. Case Studies

### 3.1. Urban Point Cloud

In order to validate the proposed methodology, we applied it to two point clouds, one corresponding to an urban area and the other to a forestry area. The first one has mainly artificial objects and was obtained using a Mobile Laser Scanner (MLS), that consists of a two-dimensional laser scanner, an Inertial Measurement Unit (IMU), and a Global Navigation Satellite system (GNSS), all of them calibrated and mounted on road vehicle. The measurement rate is 1000 points per second, and the maximum measurement range is 60 m. The vehicle drove at up to 20 km/h along 1.5 km in the CMU Campus in Oakland, USA, gathering 1.6 million points [36,37,38]. As will be shown, although the quality of the point cloud is not high, the results obtained were good, despite it being collected with a low cost system.

As can be imagined, it is essential to process these point clouds in order to select and extract the different kinds of objects. Five classes were considered: poles, ground, vegetation, buildings, and vehicles.

**Figure 1:**
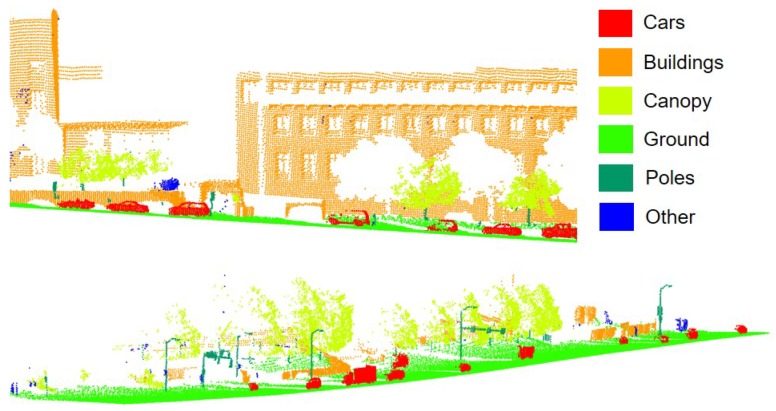
Urban dataset. The classes to be extracted are shown in color.

Figure 1 shows a small piece of the point cloud, where different colors have been assigned to each class.

**Figure 2:**
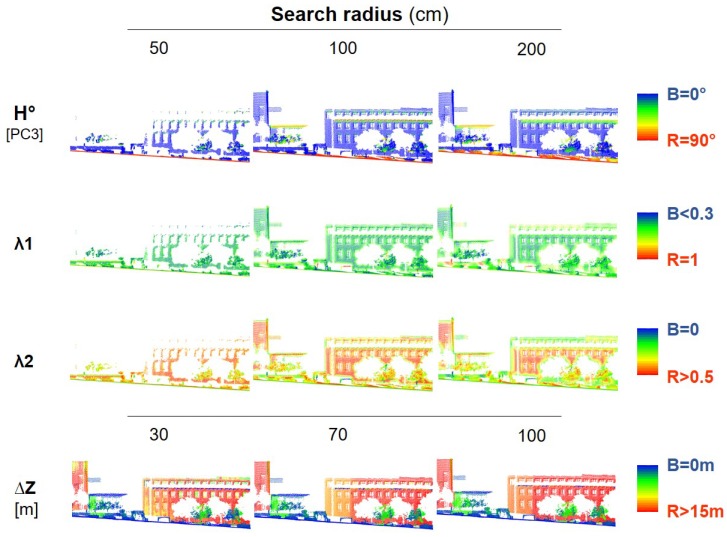
Values of λ1, λ2, horizontality, and Z range (p5–p95) at different scales for the urban dataset.

Figure 2 shows the values of the horizontality, the first two normalized eigenvalues and the Z range at three different scales. From this figure it is possible to see the influence of those features in each class. For instance, horizontal ground and vertical façades have values of the horizontality of approximately 0° and 90°, respectively.

### 3.2. Forest Point Cloud

The second point cloud tested was a forest plot, where objects to be extracted are natural. In particular, we were interested in three classes that are important in forestry: ground, trunks, and branches. Unlike the previous point cloud, the system used to perform the study was a wearable laser scanner (WLS), the ZEB-REVO (GeoSLAM) [39] mobile laser scanner. It integrates a rotating 2D scanning device, an IMU, and data storage and processing units. The system acquires information from the scanning head that is transformed into a three-dimensional point cloud by applying 3D-SLAM algorithms, instead of the integration of GNSS and IMU data performed by MLS systems. The data acquisition is performed within the default range 0.60–15 m outdoors, with a scanning rate of 40,000 points per second. The dataset contains 1.3 million points from a Sitka Spruce forest plot in Aberfoyle, Scotland (UK) that contains 10 trees. The data is owned by Forest Research (UK) and provided to the authors for the purpose of this study.

**Figure 3:**
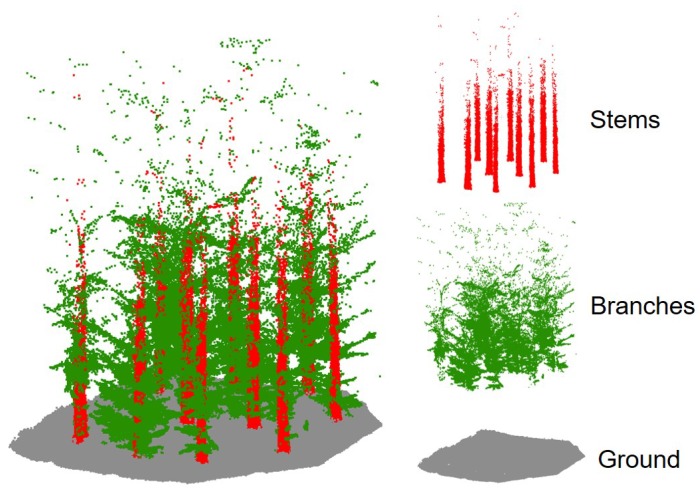
Forest dataset. The classes to be extracted are showed in color.

Figure 3 shows a small part of the forest point cloud. Points for each of the three classes extracted to train the mathematical models are depicted on the right.

**Figure 4:**
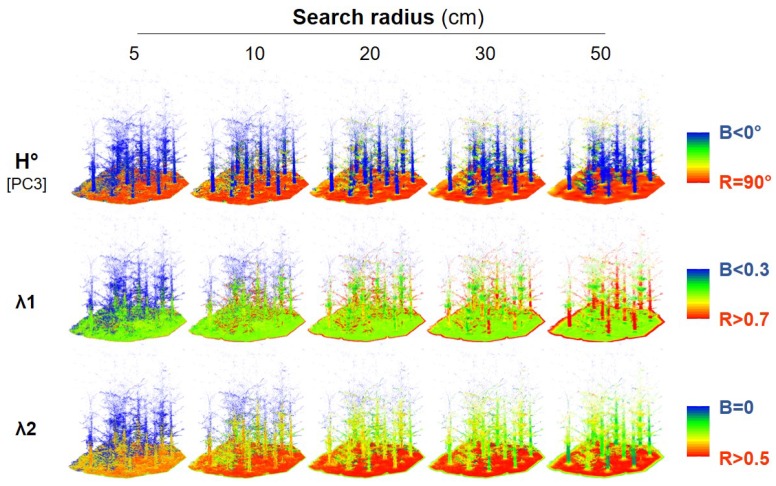
Values of λ1, λ2, and horizontality at different scales for the forest dataset.

Figure 4 shows the values of the first two normalized eigenvalues and the horizontality at five scales. As in the previous example, it is almost possible to visually distinguish the three classes from the values of these features at some scales.

## 4. Results and Discussion

The proposed methodology was applied to the data corresponding to both the urban and the forest scenarios. To construct RF and SVM models, it is necessary to fix some parameters. The same parameters were used for both datasets. For the RF model, a forest with 50 trees and a minimum and maximum of 1 and ∞ terminal nodes were fixed. For the SVM model, a radial basis function with parameter γ=0.01 and C=10 were chosen.

Table 2 shows the results for the urban dataset. Specifically, the training sample contained 20,869 observations, while the test sample had 15,553 observations. Training and test samples were independent, as they correspond to different areas without adjacent points. The metrics improve those obtained with each individual scale.

As Table 2 shows, the four models provide similar results. Given their simplicity and time of calculation, linear discriminant or logistic regression could be considered the most appropriate, but they have worse predictive performance than the other two. SVM and RF models have better behavior for almost all classes, similar to those reported in previous works, even when, mostly, the algorithms were explicitly designed to extract a specific type of object [4,9]. A review of the literature provides the following ranges of recall values: poles (0.67–0.82); vegetation (0.85–0.98); buildings (0.41–0.87); cars (0.13–0.95). However, in addition to the low quality of the point cloud, it should be taken into account that our approach does not include any preprocessing operation to remove noise and artifacts, nor color or intensity as input variables.

**Figure 5:**
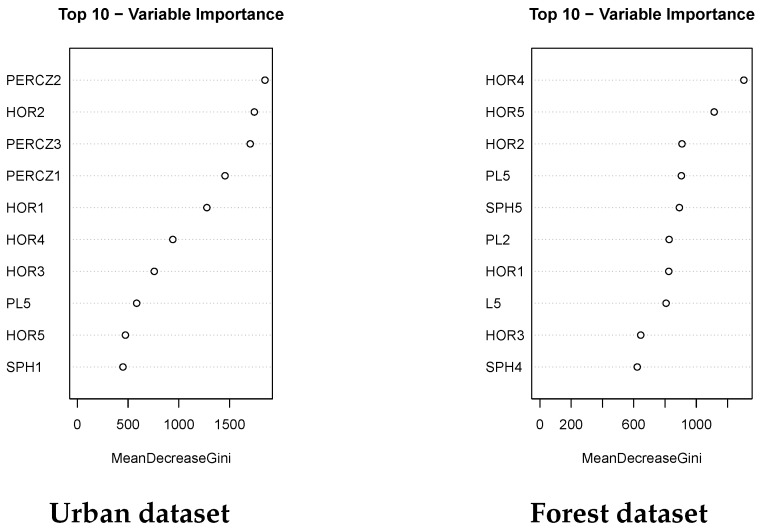
Relative importance of the top 10 input variables for the urban and the forest datasets. PERCZ, 5th–95th percentile range of the Z coordinate; HOR, horizontality; PL, planarity; L, linearity; SPH, sphericity. The number after the letters indicates the number of the scale, from 1 (5 cm) to 5 (50 cm).

For those users interested not only in prediction, but also in determining the most significant or important features, RF has methods to obtain an ordered list of them. Figure 5 (left)—provides the mean decrease in Gini coefficient for each variable, which is a measure of the variable importance. A higher mean decrease in Gini indicates higher variable importance. In this sense, the range of the Z coordinate and the horizontality are within the most important variables for the urban dataset.

Regarding the forest point cloud, the results are registered in Table 3. The size of the training and test samples were 16,603 and 7508, respectively. All models provided similar results in terms of predictive capacity, although SVM is much slower than the other three models. The three classes have high values for the five metrics, with a global accuracy for each of them above 93%. These values are really good, above those reported by other authors (see [40] for a review of forest inventory with TLS).

Again, for people interested, not only in the predictive capacity of the models but in the analysis of the features and variable selection, the RF model has the advantage of giving an ordered list of features according to its importance to discriminate between the different object categories. As in the urban scenario, according to the RF model, horizontality is among the most important variables to discriminate between the three classes (Figure 5 (right)).

## 5. Conclusions

In this work, we analyze the utility of multiscale supervised classification methods for laser scanning or photogrammetry point cloud segmentation, for both artificial and natural objects. The method relies only on the coordinates of the points (not the color or the intensity), that is, on the geometric information, which makes it independent of the system used to capture the point cloud. Only a few easily calculated variables (obtained at different scales from the coordinates) were used to solve the problem.

Four different supervised classification algorithms were tested, with behavior differing depending on the characteristics of the data. The best results were obtained for the forest dataset, reaching an overall accuracy of 96% and a Kappa coefficient of 0.93. Good metrics, although significantly lower (overall accuracy 0.85 and Kappa coefficient 0.81), were also obtained for a dataset of an urban area, despite the quality of the data. These metrics were similar and even better than those reported in previous works using different techniques.

Although similar results were obtained for the four classification methods tested, the random forest algorithm provided the best solution when accuracy, calculation time, and specification of the importance of the different input variables are considered at the same time.

Regarding the scales and the extracted features to feed the machine learning algorithms, the analysis of the relative importance of the features shows that almost all the scales contribute to the solution, and that the horizontallity is among the most important variables at different scales. In addition, we verified that a multiscale strategy superpassed a single scale strategy.

## Figures and Tables

**Table 1:** Input variables for classification.

Name	Formula
Linearity	$\frac{\lambda_{1}-\lambda_{2}}{\lambda_{1}}$
Planarity	$\frac{\lambda_{2}-\lambda_{3}}{\lambda_{1}}$
Sphericity	$\frac{\lambda_{3}}{\lambda_{1}}$
Horizontality	$\frac{acos(v_{3}\cdot z)}{∥v_{3}∥}$
Z range	$Z_{max}-Z_{min}$

**Table 2:** Metrics for the four models obtained for the urban point cloud, corresponding to the test sample.

	LR			LDA			SVM			RF		
Class	Prec	Recall	F1	Prec	Recall	F1	Prec	Recall	F1	Prec	Recall	F1
Poles	0.62	0.77	0.69	0.65	0.73	0.69	0.71	0.78	0.74	0.76	0.80	0.79
Ground	0.98	0.99	0.98	0.97	0.98	0.69	0.99	0.99	0.99	0.99	0.99	0.99
Vegetation	0.71	0.72	0.71	0.65	0.69	0.67	0.68	0.86	0.76	0.78	0.88	0.83
Buildings	0.88	0.74	0.80	0.81	0.74	0.78	0.84	0.72	0.77	0.76	0.78	0.77
Cars	0.86	0.86	0.86	0.86	0.84	0.85	0.90	0.83	0.87	0.91	0.83	0.86
Ov. Acc	0.82			0.80			0.83			0.85		
Kappa	0.77			0.75			0.79			0.81		

**Table 3:** Metrics for the four models obtained for the forestry point cloud, corresponding to the test sample.

	LR			LDA			SVM			RF		
Class	Prec	Recall	F1	Prec	Recall	F1	Prec	Recall	F1	Prec	Recall	F1
Ground	0.99	0.99	0.99	0.99	0.99	0.99	0.99	0.99	0.99	0.99	0.99	0.99
Trunk	0.86	0.89	0.88	0.89	0.86	0.87	0.89	0.98	0.93	0.91	0.95	0.93
Branches	0.94	0.95	0.95	0.94	0.94	0.94	0.96	0.95	0.93	0.95	0.96	0.96
Ov. Acc	0.94			0.98			0.99			0.99		
Kappa	0.91			0.95			0.96			0.96		
